# The contribution of distance learning to the knowledge of nursing lecturers regarding assessment of chronic wounds^1^

**DOI:** 10.1590/0104-1169.3606.2533

**Published:** 2015

**Authors:** Márcia Beatriz Berzoti Gonçalves, Soraia Assad Nasbine Rabeh, César Augusto Sangaletti Terçariol

**Affiliations:** 2 Master's student, Escola de Enfermagem de Ribeirão Preto, Universidade de São Paulo, WHO Collaborating Centre for Nursing Research Development, Ribeirão Preto, SP, Brazil; 3 PhD, Professor, Escola de Enfermagem de Ribeirão Preto, Universidade de São Paulo, WHO Collaborating Centre for Nursing Research Development, Ribeirão Preto, SP, Brazil; 4 PhD, Professor, Centro Universitário Barão de Mauá, Ribeirão Preto, SP, Brazil

**Keywords:** Pressure Ulcer, Varicose Ulcer, Foot Ulcer, Diabetic Foot, Distance Learning

## Abstract

**OBJECTIVE:**

to identify the contribution made by a refresher course on the assessment of chronic wounds, offered through the Moodle virtual learning environment (VLE), to the knowledge relating to this issue of nursing lecturers and nurses linked to higher education.

**METHOD:**

a prospective, quasi-experimental study, with data collection before and after the educational intervention. The study was undertaken in three stages using the Moodle VLE. The sample was made up of 28 participants who answered the pre-test on the knowledge, devised in accordance with international guidelines on chronic wounds. Afterwards, the refresher course was offered (intervention) and was accessed in accordance with individuals' schedules, during the established time period. At the end of the course, 26 participants answered the post-test. Those who did not participate in the post-tests were excluded from the study, as it is pairwise analysis of the sample.

**RESULT:**

the participants obtained, on average, 55.5% of correct answers in the pre-test on their knowledge, and 73.4% in the post-test, this difference being statistically significant. There was a negative correlation between the time of experience in lecturing and the performance in the test on their knowledge.

**CONCLUSION:**

the participation in the online refresher course contributed to improving the lecturers' performance in the test on their knowledge, in relation to the recommendations for assessing chronic wounds, based in scientific evidence.

## Introduction

The chronic wounds (CW), in particular pressure ulcers (PU), venous ulcers (VU) and neuropathic ulcers (NU) stand out as chronic health conditions, with great epidemiological relevance. These occurrences generate a negative impact on peoples' quality of life, and can result in prolonged episodes of inpatient treatment, an increase in morbidity and mortality, and in high social and economic costs - which constitute them as a serious public health problem^(^ 1 ^-^ 4 ^)^.

The nursing care for people with CW, based on recommendations with the best scientific evidence, requires the systematic assessment of the wound and its characteristics. This stage is the basis for decision-making and structuring the therapeutic plan, and makes it possible to monitor and document the results of the interventions, as well as the healing process^(^ 2 ^-^ 5 ^)^.

The anatomical location of the wound, the extent of the area with tissue compromise, the size of the wound, the exudative pattern, the characteristics of the tissues present in the wound bed, wound edges and adjacent skin, the bacterial load, odor and local pain constitute the aspects to be considered in assessing the wound. These characteristics provide parameters for identifying the wound's healing status^(^ 2 ^-^ 5 ^)^.

Studies have revealed gaps in nurses' knowledge regarding this issue, which needs standardization and a foundation in scientifically-based guidelines^(^ 5 ^-^ 7 ^)^. Moreover, studies involving student nurses have demonstrated that the knowledge acquired during the undergraduate course was insufficient for the future nurses to be prepared for assessing wounds and prescribing nursing interventions as part of the care actions for the person with a chronic wound^(^ 8 ^-^ 10 ^)^.

Lecturers on undergraduate courses in nursing have responsibilities in training the future nurses, which presupposes convergence between the best practices and the legal directives. This requires the explaining of the shortcomings in the teaching, and the adoption of strategies for overcoming these through constant updating and through the improvement and development of scientific knowledge^(^ 10 ^)^.

The flexibility of distance learning (DL) through virtual learning environments (VLE) is one alternative for the updating and training of professionals, which makes good use of time, and can be adjusted to the individual's routine; and has been demonstrated to be effective in improving knowledge regarding the prevention and treatment of chronic wounds^(^ 11 ^)^.

In Brazil, as in other countries of the world, the Moodle VLE has been widely used for DL. This VLE encompasses various asynchronous and synchronous resources, including chat rooms, discussion forums, blogs, glossaries, Wikipedia, an assignment submission area, files with support materials and questionnaires, among others^(^ 12 ^)^.

In the perception of nursing lecturers, undergraduate students and nurses, the Moodle VLE allows the exchanging of experiences and active discussion regarding the use of nursing practices in clinical situations, both in their formal aspects and in the aspects related to the feelings of the people involved in the care process; it is also useful as a tool for continuing education^(^ 13 ^)^.

One experimental study undertaken in Spain with 169 doctors demonstrated that using the VLE for mediating online training on palliative care, for professionals working in primary health care, was able to contribute to improving knowledge. The doctors who participated in the online educational intervention obtained an increase in their knowledge ranging from 14 to 20%. The confidence for managing symptoms and communicating increased significantly in comparison with the control group^(^ 14 ^)^.

Due to the importance of the issue referent to the knowledge of nursing lecturers for assessing chronic wounds, the study questions were: does participating in a refresher course on the assessment of chronic wounds, offered through the Moodle VLE, contribute to improving the knowledge on this issue of nursing lecturers and nurses who are linked to public and private institutions of higher education? Is there a correlation between the demographic and academic profile of the participant and the level of knowledge for assessing chronic wounds, before and after the educational intervention?

The objective of the present study was to evaluate the contribution of distance learning for increasing the knowledge of nursing lecturers, and nurses linked to teaching, in public and private higher education institutions in a municipality in the non-metropolitan region of the State of São Paulo, regarding the assessment of chronic wounds. 

## Methods

This prospective, quasi-experimental study was approved by the Research Ethics Committee of the institution to which the researcher was linked, and respected all the ethical principles pertinent to the investigation, receiving the Certificate for Presentation for Ethical Appreciation N. 02158012.5.0000.5393.

The sample was made up of 28 nursing lecturers and nurses linked to teaching, from the undergraduate courses in Nursing of two Higher Education Institutes (HEI) of a municipality in the non-metropolitan region of the State of São Paulo. 

The inclusion criteria for participating in the study were to be a nurse and to work in lecturing in higher education in theoretical and/or practical courses. The exclusion criteria were to not have access to the Internet and not to undertake the post-test, as it was a pairwise analysis of the sample.

The educational intervention, the independent variable of this study, was offered as a Distance Learning refresher course, titled "Assessment of chronic wounds in nursing care", which was produced and validated^(^ 15 ^)^ in accordance with the WOCN (Wound, Ostomy and Continence Nurses Society), for caring for people with pressure ulcers, venous ulcers, and neuropathic ulcers^(^ 2 ^-^ 4 ^)^, and was adapted for the present study's population. 

The course was available from May - August 2013 on the Moodle virtual learning environment, with an hourly workload of 05 hours.

Data collection, referent to the dependent variable (the lecturers' knowledge regarding the issue, correlation between the participants' academic profile and their performance in the test on their knowledge) occurred in two stages: before and after their participation in the course. Initially, as a prerequisite for accessing the course content, the participants undertook a pre-test, administered virtually, using the Moodle VLE; after the end of the activities proposed, the post-test was made available, individually, to each participant. 

Adding the module using the Moodle VLE was undertaken using the Flash Player*(r)* program, to stop the material offered being downloaded and printed, and to block consultation of the educational module during the undertaking of the post-test. In this stage of the collection, 26 participants answered the test on their knowledge. The other two participants neither concluded the intervention nor answered the post-test, and were excluded from the sample. 

The data collection instrument was structured in two parts. Part I contained 14 questions adapted from the instrument used by Miyazaki, Caliri and Santos (2010), for the population of this study, which sought to identify the demographic and academic characteristics of the participants, such as time of experience in teaching, the area in which the person worked, and search strategies adopted for updating knowledge. 

Part II of the data collection instrument was a test on knowledge, with thirty questions on the assessment of characteristics of chronic wounds, categorized in five domains of knowledge, these being: "etiology" (06 questions), "dimensioning" (07 questions), "wound bed" (08 questions), "edge and peri-wound skin" (03 questions) and "infection" (05 questions); the participant had to choose between the alternatives "true", "false" and "don't know". These questions were elaborated based on the recommendations of the WOCN international guidelines^(^ 2 ^-^ 4 ^)^, and were validated by specialist judges and experts in the issue in relation to their clarity, ease of comprehension, language used and relevance of the question. The instrument was adjusted in accordance with the validators' suggestions. 

The participants' demographic and academic data were described through frequency distribution (absolute and relative), mean values and the respective standard-deviations (SD), represented through tables and graphs. In order to evaluate the participants' performance before and after the intervention, the Kolmogorov-Smirnov test was administered in order to ascertain the normality of the variables of the participants' knowledge before and after the educational intervention. As the data passed this test of normality, they were compared using the Student two-tailed paired parametric t-test, in order to compare the means of the number of correct answers, of errors and of "don't know"s in the pre- and post-test, for the domain of knowledge, and for the correlations. In order to compare the number of correct answers in the pre- and post-test, by question, the non-parametric Wilcoxon paired test was administered. The Pearson correlation index was calculated between the participants' age and their performance in the pre- and post-test, as well as their time of experience and performance in the pre- and post-test.

In order to ascertain if there was a significant difference in the number of correct answers among the lecturers/nurses who participated in events related to the issue, information was exchanged regarding the issue among peers both in- and outside the HEI, and they sought to update their knowledge using the Internet, and the non-paired two-tailed Student t-test was administered to those who stated that they did not. 

## Results

The mean age among the study participants was 42.3 years (SD 9.56) and all were female. The mean time of experience in teaching was 11.16 years (SD 8.02); the participant with the least time of experience had not completed one year in teaching, while the participant with the longest time of experience had worked in the area for 31 years. Both were from a public HEI. 

The majority worked in courses in the areas of clinical nursing and/or nursing administration (88.5%), and a large proportion worked in collective health (42.3%). For the teaching of the clinical practice, 61.5% stated that they worked in hospital care, 15.4% in primary care and 23.1% in both areas. In these scenarios, 91.7% mention providing care to people with chronic wounds, in the care ambit, in the managerial ambit, or in both. None of the participants were specialized in the area in question. 

For updating their knowledge, the main strategies mentioned by the participants were searching for information on chronic wounds with other professionals from the teaching institution (65.4%) or from outside it (61.5%), and from the Internet (73.1%). 

Prior to the educational intervention, the mean for correct answers in the pre-test was 55.5%, with the worst performance for the domains of "etiology" and "infection", with 48.3 and 47.9% of correct answers, respectively. The domain of "dimensioning" obtained the largest number of questions filled out correctly (65.3%).

It was identified that 25 (96.1%) participants correctly answered less than 70% of the test on their knowledge, it being the case that 7 (26.9%) correctly answered fewer than half, only 1 of them (3.8%) correctly answered up to 79.1% of the questions, and no participants scored 80% of correct answers or more.

**Figure 1 -:**
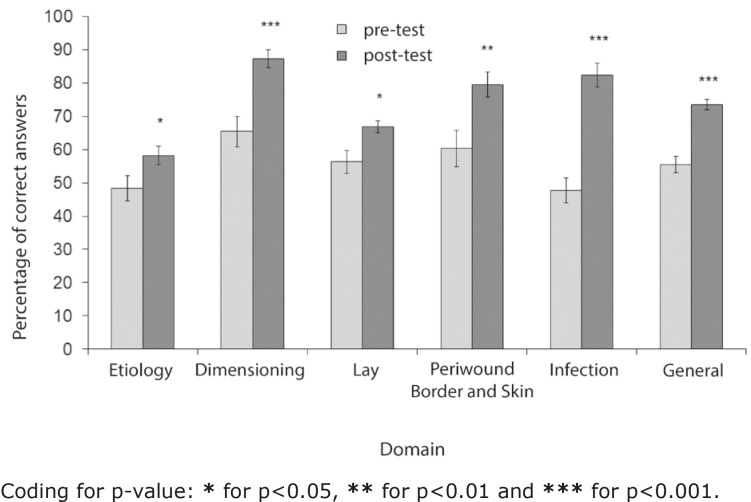
Proportion of correct answers in the pre- and post-test for each domain of knowledge. Ribeirão Preto, State of São Paulo (SP), Brazil, 2013

The participants' performance improved following the intervention, in that the mean for correct answers was 73.4%. None of them provided correct answers to fewer than half of the questions, 10 (38.4%) obtained between 70 and 79.9% correct answers, and 9 (34.6%) correctly answered over 80% of the questions, the highest percentage of correct answers being 86.6%. A statistically significant increase was observed in the percentage of correct answers, for each domain of knowledge, individually, as well as for general knowledge, in the post-test (Figure 1). 

The number of correct answers per question was greater after participating in the course, for 76.6% of the participants. The domains of "infection" and "dimensioning" obtained the best performance in the post-test, with a significant increase in the number of correct answers for the majority of the questions in these domains, according to the Wilcoxon test.

Three questions from the domain of "etiology" which dealt with etiological factors of the PU and of the definition of stage I of this ulcer obtained low performance in the pre- and post-test. 

The domain of "infection" obtained a higher percentage of correct answers in the post-test for all the questions, in comparison with the other domains (mean of correct answers in the post-test, 82.3%). 

According to the Pearson correlation index, there was a negative correlation for the participants' time of experience and the number of correct answers in the pre- (r=-0.06845) and in the post-test (r=-0.5330), a statistically significant finding in the post-test (p<0.01), with a worsening in the performance in the test on knowledge, proportional to the increase in the time of experience. 

There was a statistically significant improvement in the participants' performance after the intervention, both for the group of participants who stated that they undertook activities associated with "chronic wounds", in the practice of teaching (p=0.00001) and used some strategy for seeking information on the issue - Internet (p=0.000003), the exchanging of information between peers (p=0.0001), participation in scientific events issue (p=0.004) - and for the group which mentioned not doing this (p=0.001). For the first group, the mean percentage of correct answers was 57.5% in the pre-test, and 73.1% in the post-test. For the second group, the mean percentage of correct answers was lower in the pre-test (51.1%), although in the post-test, the mean obtained was identical to that of the first group. 

The group which stated that it did not adopt strategies for updating knowledge, in comparison with the group which stated that it did so, obtained a greater increase in the level of knowledge from the pre- to the post-test, that is, the internal gain was greater. The comparison between the differences was statistically significant (p=0.048) only for the strategy of "seeking information among peers from the same HEI". 

## Discussion

Knowledge gaps among nurses and nursing professionals in relation to the assessment and treatment of chronic wounds are reported in the Brazilian and international literature, which emphasizes that in spite of the professionals' awareness regarding the issue, the care provided diverges from the recommendations grounded in scientific evidence, with the adoption of empirical practices, which corroborates the present study's results and points to the need for updating the health professionals through continuing education^(^ 16 ^-^ 18 ^)^.

In their current work, in relation to the knowledge regarding the classification of the PU in "stage I - non-blanching hyperemia", the participants presented poor performance both before and after the educational intervention. A similar result was observed in an experimental study held in order to identify the knowledge of nurses and student nurses regarding the classification of PU, in which stage I of the PU was erroneously classified as "blanching erythema" by the majority of the participants^(^ 19 ^)^.

Not knowing the correct definition and characteristics of stage I of the PU results in late intervention against the worsening of the PU, although this stage can indicate lower severity without underlying tissue damage, and can be reversed^(^ 19 ^)^. The lecturer needs to direct the practice of teaching, emphasizing the commitment which the nurse has in relation to patient safety and that, for this, the nurse needs to be prepared to provide up-to-date knowledge. 

Also observed was poor performance in the test on knowledge in relation to the etiology of chronic wounds. The literature emphasizes that restricting the care provided to the person with a chronic wound to topical therapy and failing to take into account the etiological factors makes the therapeutic plan inefficacious and impedes the complete healing of the wound^(^ 2 ^-^ 4 ^)^.

The refresher course entitled "Assessment of chronic wounds in the nursing care" had a positive impact on the participants' performance, with a significant increase in the correct answers following the educational intervention. This finding was similar to that of another work^(^ 20 ^)^, which sought to identify the effect of an online course regarding prevention and treatment of PU, available on the Moodle VLE, on the knowledge of nurses working in an ITU unit, in a hospital in Fortaleza in the Brazilian state of Ceará.

The domain of "infection" obtained a statistically significant reduction in the number of errors, which points to the educational model's contribution to the dissemination of the recommendations of the international guidelines regarding the management of the infected wound. Infection is one of the factors which most frequently impedes or delays healing. In order to direct the treatment, tissue biopsy is considered the gold standard in distinguishing between contamination, critical colonization, and infection^(^ 2 ^-^ 4 ^)^.

This work found a negative correlation between the time of experience in lecturing and the performance in the test of knowledge. Similar results have been found in other works^(^ 6 ^,^ 21 ^)^. In recent decades, the production of knowledge, and the development of new technologies for caring for people with chronic wounds have presented important advances. This new condition points to the nursing professionals' need for constant and scientifically-based updating, whether in the direct provision of care or in teaching, although time of experience is a fact which can contribute to improving practices and knowledge^(6,10).^

Nevertheless, the experience in lecturing should promote the improvement of the practicing of teaching, given that it provides opportunities both for the process of learning knowledge and for developing teaching skills^(^ 10 ^)^.

This study made it possible to infer that seeking information on "chronic wounds", through other strategies, was associated with participants' better performance in the test of their knowledge, before the educational intervention. Brazilian and international works are relevant to the findings of the present study, as the search for updating is essential for maintaining knowledge, whether through updating through the scientific knowledge available in media for the diffusion of knowledge, such as the reading of scientific articles, the use of the Internet and/or library, participation in scientific events and others, or through specialized professional qualification^(^ 6 ^,^ 22 ^)^.

Based on the presupposition that updating is fundamental and that the lecturer on undergraduate courses in Nursing has responsibilities in the training of future professionals, it is essential to consider the gaps in the professors' training as a problem which generates reflections and gives rise to initiatives with efforts to close them^(^ 17 ^)^.

The attitude of the nursing lecturer needs to be aligned with public health policies, which require critical, reflexive and active professionals who are committed to the quality of the care provided. To this end, the practice of higher education requires a constant search for the development of critical and scientific knowledge, and encompassing reflection on this knowledge's contribution in the construction of society^(^ 10 ^)^.

The literature has indicated the intensification of the workload of nursing lecturers in higher education, which exceeds the workday and extends into the domestic environment, with requirements for complying with productivity goals, which has resulted in work overload and dissatisfaction, and has been implicated in illness^(^ 23 ^)^. These conditions can compromise the lecturers' participation in activities of updating knowledge which require time. 

In this perspective, distance learning is configured as a strategy for updating knowledge which makes good use of time, due to the flexibility which it allows people, as they choose when and where to access it. The dynamic architecture of the VLE makes it possible to rapidly access new information, which contributes to nurses' knowledge not becoming out of date. Besides this, the virtual environment makes it possible to compile massive quantities of information, and provides access to a variety of scientific documents^(^ 11 ^)^.

Nevertheless, it is important to consider that continuing education is necessary, although it may be insufficient for causing changes in the practice. The literature indicates that in addition to acquiring scientific knowledge, other factors, such as a context which is favorable to consolidating evidence-based practice (EBP), and the professional's perception regarding the importance of adopting scientific evidence in order to qualify the care, can also influence the decision to abolish the use of practices which are not evidence-based^(^ 17 ^,^ 24 ^)^.

## Conclusion

Knowledge grounded in the best scientific evidence for providing nursing care to people with chronic wounds must guide the teaching of the issue in the training of students of Nursing, and discourage the reproduction of practices which are not evidence-based, rooted only in professional traditions.

This result reaffirms that the knowledge regarding the assessment of chronic wounds must be considered as essential knowledge by all nursing lecturers, as these face the challenge of leading the learning for the care for people with this complication, in the varying contexts of healthcare.

Following the educational intervention, there was a significant improvement in the participants' general performance, in each one of the domains of knowledge tested. This denotes that participation in the virtual updating course for assessment of chronic wounds for the nursing care, offered through the Moodle VLE, had a positive impact on the knowledge of the nursing lecturers and nurses linked to higher education. Bearing in mind that the above-mentioned course was developed based in the recommendations publicized through the guidelines of the Wound, Ostomy and Continence Nurses Society, it is considered to be useful in the dissemination of scientific evidence, for the assessment of chronic wounds. 

In the light of the importance of understanding the wound development process for caring for people with chronic wounds, and of the participants' poor performance in the test on knowledge relating to the ulcers' etiology, it is believed that the knowledge regarding the etiology and physiopathology of chronic wounds, made available in the support material, even though as optional material, should be part of the unit as mandatory reading as a basis for knowledge regarding wound assessment. 

The results also allow one to infer that distance learning can be an effective strategy for updating knowledge for lecturers from various areas of teaching and contexts of care, given that all gained from the intervention. 

This study supports reflections regarding the impact of the work of the lecturer who seeks updated knowledge based in the best scientific evidence on the training of new nurses and - as a consequence - on the quality of the care provided to the person with a chronic wound.

The efforts to improve the practice of teaching on undergraduate courses in Nursing, in relation to the assessment of chronic wounds, do not finish with the dissemination of the best scientific evidence, but, rather, raise the challenge of seeking to investigate other strategies, such as the use of the VLE for transferring knowledge. 
